# Protein Homeostasis Networks and the Use of Yeast to Guide Interventions in Alzheimer’s Disease

**DOI:** 10.3390/ijms21218014

**Published:** 2020-10-28

**Authors:** Sudip Dhakal, Ian Macreadie

**Affiliations:** School of Science, RMIT University, Bundoora, Victoria 3083, Australia; sudip.dhakal@rmit.edu.au

**Keywords:** Alzheimer’ disease, yeast, proteostasis, unfolded protein response, autophagy, ubiquitin proteasome system

## Abstract

Alzheimer’s Disease (AD) is a progressive multifactorial age-related neurodegenerative disorder that causes the majority of deaths due to dementia in the elderly. Although various risk factors have been found to be associated with AD progression, the cause of the disease is still unresolved. The loss of proteostasis is one of the major causes of AD: it is evident by aggregation of misfolded proteins, lipid homeostasis disruption, accumulation of autophagic vesicles, and oxidative damage during the disease progression. Different models have been developed to study AD, one of which is a yeast model. Yeasts are simple unicellular eukaryotic cells that have provided great insights into human cell biology. Various yeast models, including unmodified and genetically modified yeasts, have been established for studying AD and have provided significant amount of information on AD pathology and potential interventions. The conservation of various human biological processes, including signal transduction, energy metabolism, protein homeostasis, stress responses, oxidative phosphorylation, vesicle trafficking, apoptosis, endocytosis, and ageing, renders yeast a fascinating, powerful model for AD. In addition, the easy manipulation of the yeast genome and availability of methods to evaluate yeast cells rapidly in high throughput technological platforms strengthen the rationale of using yeast as a model. This review focuses on the description of the proteostasis network in yeast and its comparison with the human proteostasis network. It further elaborates on the AD-associated proteostasis failure and applications of the yeast proteostasis network to understand AD pathology and its potential to guide interventions against AD.

## 1. Introduction

**Figure 1:**
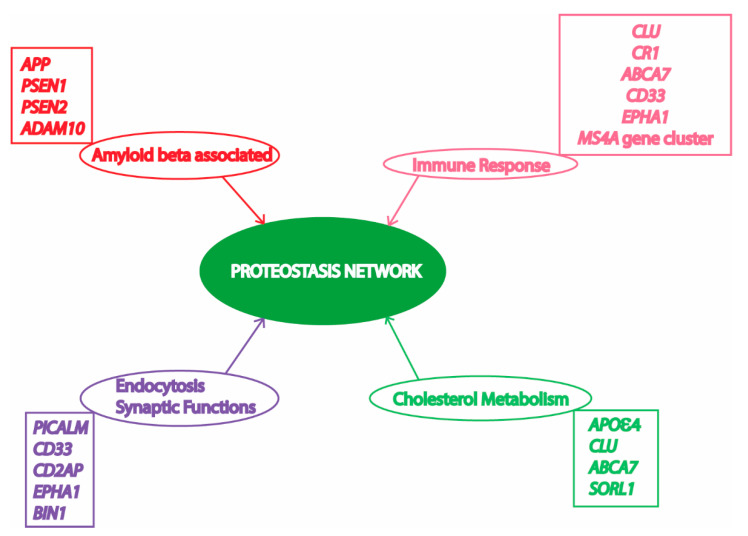
Risk factors/loci and associated cellular processes identified by genome wide association studies (GWAS) involved in Alzheimer’s Disease (AD) converge to the proteostasis network. APP, amyloid precursor protein; PSEN1, presenilin 1; PSEN2, presenilin 2; ADAM10, ADAM metallopeptidase domain 10; CLU, clusterin; CR1, complement C3b/C4b receptor 1; ABCA7, ATP binding cassette subfamily A member 7; CD33, CD33 molecule; MS4A, membrane spanning 4-domains A; PICALM, phosphatidylinositol binding clathrin assembly protein; CD2AP, CD2 associated protein; EPHA1, EPH receptor A1; BIN1, bridging integrator 1; APOε4, apolipoprotein E4; SORL1, sortilin related receptor 1. Various risk loci have been identified by GWAS to be associated with AD. Some of these risk loci are clustered to important cellular processes in this figure to depict their relationship with the proteostasis network, highlighting its significance in AD pathology. Previous studies suggest an important role of the proteostasis network in cellular processes associated with AD, including amyloid formation pathway, immune response mechanisms, vesicle trafficking, endocytosis, and lipid metabolism.

Alzheimer’s disease (AD) is an age-related progressive neurodegenerative disorder, which accounts for 60 to 80% deaths resulting from dementia in elderly people [1]. AD has been found to be a multifactorial disease with several causes being hypothesized and investigated [2]. However, amyloid beta (Aβ) mediated toxicity and its clearance from the neuronal environment has been the most popular area of AD research for the last three decades, as amyloid plaques are the most significant pathological hallmarks of the disease [3]. Memory deficits and cognitive impairment due to loss of neurons resulting in cortical atrophy accompanied by presence of amyloid plaques and neurofibrillary tangles are present in people with AD [4,5]. Depending upon the time of occurrence of first symptoms, AD victims have been classified into early onset AD (EOAD) and late onset AD (LOAD). Most AD patients (90–98%) have late onset disease with symptoms appearing after the age of 65 years [5]. AD pathology has been divided into familial autosomal dominant inheritance forms (<1% of total AD cases) and a sporadic multifactorial category (majority of cases) [6]. The mutations in amyloid precursor protein (APP) and two presenilin genes (PSEN1 and PSEN2) have been found to be associated with the familial form [7]. On the contrary, the multifactorial sporadic disease is more genetically complex, and cannot be attributed by any single risk factor or cause. More than 20 risk loci have been identified from previous studies involving genome wide association studies (GWAS) and linkage analysis (Figure 1). Most of these important AD-associated risk loci are associated with Aβ mediated toxic pathways, while other risk loci are associated with immune function, endocytosis, synapsis, and lipid metabolism [8]. Interestingly, all these cellular processes associated with AD pathology are somehow dependent on cellular protein homeostasis [9,10,11,12,13].

The intracellular neuronal environment of AD patients is characterized by loss of proteostasis, mitochondrial dysfunction, impaired mitophagy, oxidative damage, genetic instability, impaired protein clearance, amyloidosis, loss of synapsis, disruption of lipid homeostasis, biometal distribution alteration, and energy failure [2]. Most of these defects are found associated with various events in the AD brain; however, the majority are thought to be the consequence of Aβ overproduction and aggregation [14]. These intracellular cues are also conserved in yeast cells with more than 60% of its genes having human homologs or at least one conserved domain [15]. The major yeast models used for AD studies involve *Saccharomyces cerevisiae* [16]. These models for AD studies have been very important in understanding the role of conserved fundamental eukaryotic processes in AD pathology to develop methods to find potential therapeutics [17,18,19,20,21,22,23]. The conservation of signal transduction, energy metabolism, proteostasis network, lipid metabolism, vesicle trafficking, oxidative phosphorylation, stress response, longevity, and cell death in yeast models makes it highly appropriate for studies on AD pathology and treatment [15,24]. Various yeast models have been used in the past to understand AD progression and to find interventions to prevent and cure the disease [25]. This review reiterates on the comparison of the yeast protein clearance mechanisms with human counterparts and hints at the application of yeast to address important aspects of AD pathology and drug discovery.

## 2. Protein Homeostasis Network in Yeast

**Figure 2:**
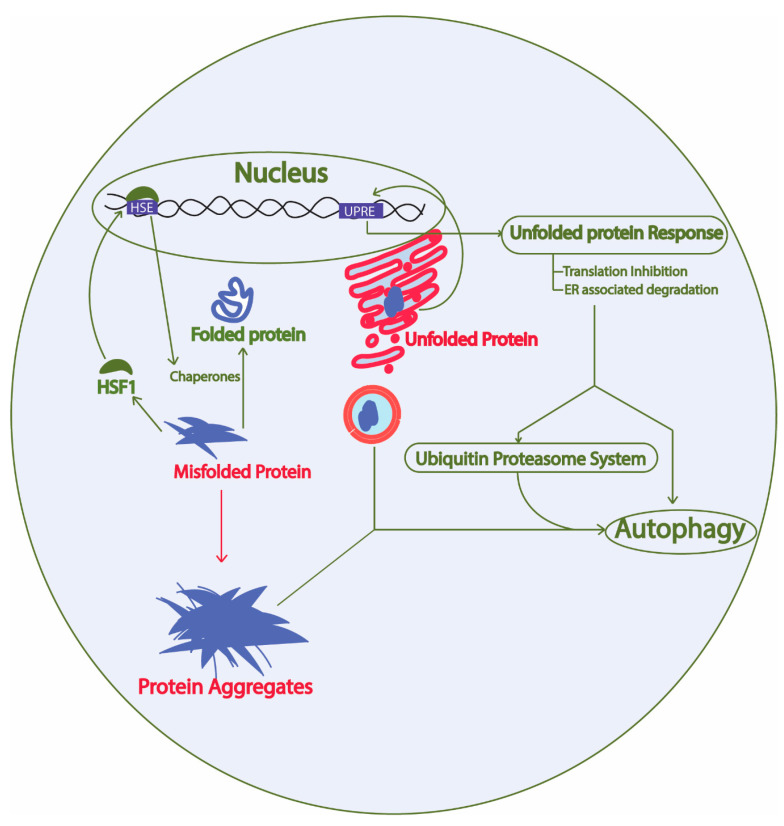
Proteostasis network involves protein synthesis, protein folding, heat shock response, unfolded protein response, ubiquitin proteasome system, and autophagy. Green arrow, protective; Red arrow, toxic; HSF1, heat shock factor 1; HSE, heat shock elements; UPRE, unfolded protein response elements. The proteostasis network involves protein synthesis machinery, protein folding, protein quality control, protein transport, and the overall turnover of proteins inside a cell. The presence of misfolded proteins in the cytosol trigger HSF1 activation, which activates promoters with HSE and results in expression of heat shock proteins that are either involved in protein disaggregation and refolding or clearance of the unwanted proteins through protein quality control mechanisms. Similarly, the presence of aberrations in the ER lumen causing unfolded protein formation leads to activation of the unfolded protein response and downstream activation of the protein quality control system.

Constant turnover of cellular components inside a cell is of the utmost importance for adaptation during changing cellular environment, which interplays with multiple cellular processes required for cell survival [26]. Numerous proteins are coordinately functioning in a cell to fulfill the survival requirements of the cell. Protein synthesis, proper folding, protein quality control, transport of the protein to the target location, and proper function of the protein in its target depends on the cellular environment. Defects in protein processing or folding can cause abnormal aggregation of proteins possibly leading to disease if not repaired. These aberrations are detected by the cell and repaired by the protein quality control mechanisms governed by the proteostasis network. In fact, at least two types of chaperones in a eukaryotic system are controlling the protein folding: chaperones linked to protein synthesis (CLIPS) and chaperones expressed during stress [27]. In eukaryotes, chaperone mediated protein disaggregation machinery allows disaggregation of the protein aggregates and helps to correct the misfolding or partial unfolding of the protein [28]. However, failure to fold the proteins in correct conformation and inability of the chaperones to refold the aggregated proteins leads to activation of protein clearance mechanisms. Generally, protein clearance in the eukaryotic cell is controlled by three major molecular pathways: unfolded protein response, ubiquitin proteasome system, and autophagy (Figure 2) [29]. Various intrinsic and extrinsic factors play a crucial role in the regulation of these pathways inside a cell. Ageing has been found to be one important reason for the loss of proteostasis and subsequent disease development in neuronal cells due to accumulation of unwanted misfolded proteins [30]. During AD pathology, deposits of amyloid plaques and neurofibrillary tau tangles are the evidences that support the loss of protein homeostasis as leading cause of the disease [31]. Restoring the protein homeostasis has been hypothesized to cure or prevent AD and similar diseases [32]. This review elaborates on molecular mechanisms of unfolded protein response, autophagy, and ubiquitin proteasome system in yeast and their relevance to study human counterparts in relation to AD progression. Furthermore, we suggest possible applications of the knowledge of proteostasis network originating from yeast to find ways to explore AD pathology and develop drugs.

### 2.1. Unfolded Protein Response Conserved in Yeast

Production of a protein is not sufficient for the proper biological function of protein; the expressed protein needs to be folded in its native form to be functional [33]. Most soluble proteins are folded directly in cytoplasm with the help of molecular chaperones [34], while the secreted proteins including hydrophobic membrane proteins pass through the endoplasmic reticulum (ER) and Golgi complexes and require correct folding [35]. Correct folding and maturity of the protein depends highly on the nutrient conditions, ER calcium homeostasis, availability of molecular chaperones, redox status, and cell health [36]. However, failure to maintain cellular homeostasis results in unfolded and misfolded proteins that are prone to aggregation. The formation of unfolded and misfolded proteins in the ER lumen triggers proteotoxic stress and activates the stress response pathway—the unfolded protein response (UPR) [37]. UPR is a mechanism by which the cells reduce the proteotoxic stress generated in the ER during protein folding and processing [36]. At least two types of stress response are activated during such conditions in mammalian cells. At first, the UPR attenuates protein translational activity by phosphorylating the translational initiation factor 2α (eIF2α) and activating the mRNA decay mechanism [38]. On the other hand, it increases protein folding, and activates autophagy and the ubiquitin proteasome system for degradation of undesired protein products [39]. On the other hand, UPR also activates the oxidative stress response to aid cell survival under ER stress [40].

#### 2.1.1. Yeast Unfolded Protein Response

**Figure 3:**
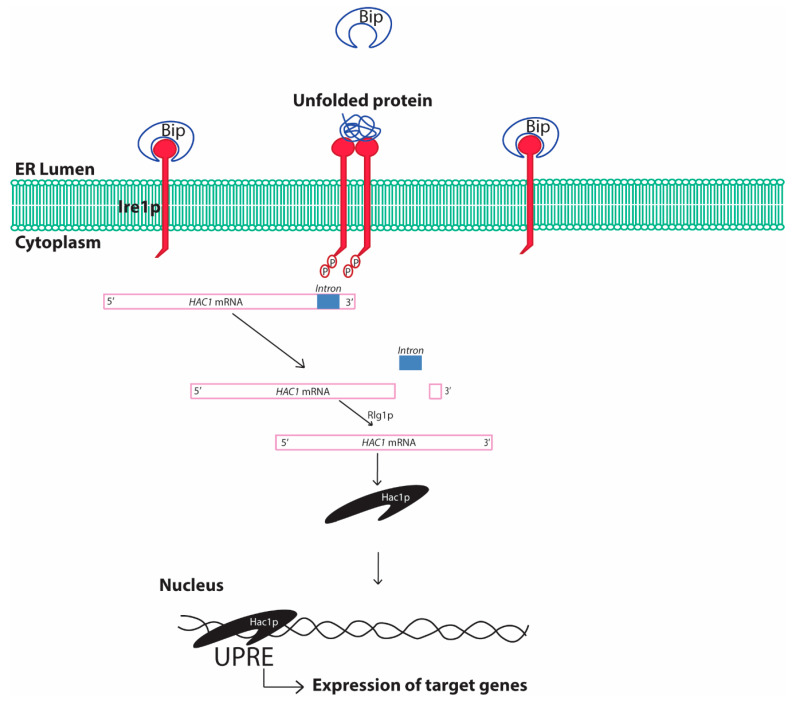
Schematic diagram showing unfolded protein response in yeast cells. Bip, binding immunoglobulin protein; ER, endoplasmic reticulum; Ire1, inositol requiring element 1; HAC1, Homologous to ATF/CREB1; Rlg1p, tRNA ligase protein; UPRE, unfolded protein response elements. The presence of unfolded/misfolded proteins in the ER lumen is recognized by the yeast Ire1 sensor protein that activates the specific cytosolic endonuclease activity cleaving the inhibitory intron of HAC1 mRNA and rendering HAC1 mRNA translationally active. Hac1p protein translates and translocates to the nucleus, where it recognizes the characteristic cis-acting elements in the promoter regions of certain genes referred to as UPRE and increases expression of genes under control of such promoters. The process is referred to as the UPR.

The UPR is conserved from the simplest eukaryotic unicellular yeast to complex human beings [36]. In yeast, the UPR involves sensing of unfolded proteins with the help of the stress sensor inositol requiring element 1 protein (Ire1p) in the ER lumen (Figure 3). The Ire1p is an ER localized type I transmembrane protein with its luminal domain sensing the protein folding environment in the ER and its cytosolic part comprising protein kinases and RNases activity. The luminal portion of the Ire1p is constantly bound with the chaperone binding immunoglobulin protein (Bip/GRP78) in normal conditions hindering the activation of Ire1p [41]. Any aberrations in the protein folding environment is indicated by the presence of unfolded and misfolded proteins in the ER or disassociation of Bip from Ire1p luminal part. The binding of Bip with Ire1p plays a crucial role in regulating UPR in yeast [42]. The oligomerization of Ire1p followed by its autophosphorylation is an essential activation signaling process that activates the cytosolic Ire1p RNase activity. The activation of Ire1p RNase activity leads to *HAC1* (homologous to ATF/CREB1) mRNA splicing [43]. In normal conditions, *HAC1* mRNA contains an inhibitory intron at the 3′ end, which renders *HAC1* mRNA unfavorable for translation and remains inhibited by yeast protein two 1 (Ypt1p) [44]. Upon ER stress, the RNase activity of cytosolic part of Ire1p cleaves the specific inhibitory intron present in the 3′ end of the *HAC1* mRNA. Moreover, re-joining of the cleaved active *HAC1* mRNA ends is accomplished by tRNA ligase (Rlg1p) protein [45]. This processing of the *HAC1* mRNA leads to its higher translation to Hac1p protein. Hac1p, protein encoded by the translationally active *HAC1* mRNA, is a basic leucine zipper (bZIP) transcription factor that recognizes specific unfolded protein response elements (UPRE) sequences (5′-CACCTTG), present in the promoters of target genes including the *Hac1* gene. Although Hac1p is sufficient for activation of UPR in yeast, complex interaction with other transcription factors involving interplay with Hac1p may result in activation of the UPRE promoters [46]. The upregulation of the promoters with UPRE region and subsequent UPR are protective to cells leading to the degradation of the unfolded protein by activating ER associated degradation (ERAD) [47]. The expression of stress resistance genes, autophagy genes and restoration of the energy metabolism further enhances cell survival. In addition, ER stress also reduces protein synthesis by inhibiting the eukaryotic translation initiation factor, which has been reported to be involved in assembly of mRNA-protein (RNP) granules [48]. At least two types of RNP granules, namely processing bodies (P-bodies) and stress granules, are found to be produced during various stresses including ER stress in yeast [49]. Formation of these granules, specifically the stress granules, are typically cytoprotective in nature and is an integral part of protein quality control.

#### 2.1.2. Human Unfolded Protein Response

The human homologs of Bip, Ire1p, and Hac1p share high similarity in their molecular mechanism and the activation of important cytoprotective processes in mammalian cells, providing a rationale to explore yeast as model organism to study UPR and ER stress (Table 1). However, the mammalian UPR is more complex than the yeast UPR. The mammalian UPR is accomplished by three mechanisms dependent on three sensors, namely, inositol requiring element 1 (IRE1), PKR-like ER kinase (PERK), and activating transcription factor (ATF6), in the ER lumen [50]. The conserved *IRE1* signaling in mammals causes the splicing of X-box binding protein 1 (XBP1, the Hac1p functional homolog in mammals) transcription factor mRNA and downstream signaling [51]. Additionally, the PERK protein is similar to Ire1p of yeast in the luminal part, while it is different in the cytosolic part. The activation of PERK leads to activation of the protein kinase domain that phosphorylates the eukaryotic translation initiation factor 2 alpha (eIF2α), which reduces the protein translational activity [52]. In humans as well, the inhibition of protein translation is associated with the formation of RNP granules, which is generally cleared by either protein disaggregation machinery or by autophagy [49]. In contrast, ATF6 sensors are absent in yeast and function very differently from the conventional response. Upon ER stress, ATF6p is transported to Golgi complexes, where it is cleaved by two proteases, specificity protein 1 (SP1) and specificity protein 2 (SP2), to release a fragment ATF6f. The ATF6f fragment translocates to the nucleus and acts as a bZIP transcription factor, which binds to the DNA in the promoter region of the target genes involved in ER associated degradation (ERAD) [52]. Meanwhile, the failure to protect cells from chronic ER stress may result in the activation of activating transcription factor 4 (ATF4)/C/EBP homologous protein (CHOP) signaling. The ER stress-induced CHOP signaling activates proapoptotic genes and reduces antiapoptotic gene expression [53]. Simultaneously, CHOP signaling also upregulates expression of growth arrest and DNA damage 34 (GADD34p) protein phosphatase, which dephosphorylates eIF2α enhancing protein synthesis, thereby inducing formation of reactive oxygen species as a result of compromised ER folding environment and chronic ER stress [54,55]. The overall effect of chronic ER stress is characterized by the activation of programmed cell death via apoptosis [56].

### 2.2. Ubiquitin Proteasome System

The ubiquitin proteasome system (UPS) is one of the major protein quality control systems that controls intracellular protein homeostasis by facilitating degradation of unwanted proteins [61]. The presence of the UPS in both the cytosol and nucleus indicates its significance during transcriptional and post-translational regulation of genes. The UPS plays a significant role in neuronal synapsis by regulating the DNA damage repair, neurotransmitter release, and membrane receptor turnover [62,63,64]. The degradation of protein due to UPS is dependent on the cellular processes and energy status. In nutrient rich conditions, the availability of energy helps proteasomal degradation of polyubiquitinated target proteins, while nutrient starvation lowers the cellular energy levels, which may restrict the UPS and enhance autophagy to save energy [65].

**Figure 4:**
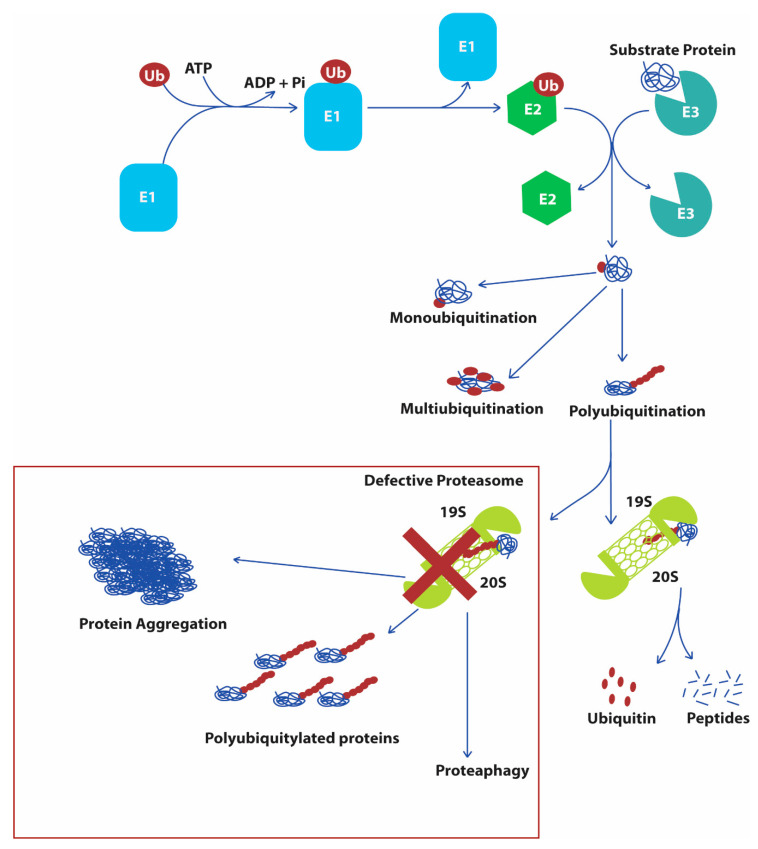
Schematic diagram showing degradation of substrate protein by the UPS and fates of defective proteasomal system. Ub, ubiquitin; ATP, adenosine triphosphate. The first step in ubiquitin proteasome system requires the activated E1 activating enzyme to activate the ubiquitin. Following the activation of ubiquitin, E1 enzyme transfers the activated ubiquitin molecule to E2 conjugating enzyme. At the same time, E3 ligating enzyme conjugates with the substrate protein, which is then conjugated with ubiquitin bound to E2 enzyme. The chain of ubiquitin is elongated by multiple cycles of ubiquitination. The ubiquitinated proteins are then recognized and degraded by the 26S proteasome, while the failure to degrade the polyubiquitinated proteins due to defective proteasome may result in protein aggregation, abundance of polyubiquitinated proteins, and impaired protein turnover. The defective proteasome may get recycled through proteaphagy.

Protein turnover through the UPS occurs through ubiquitin-mediated proteasomal degradation (Figure 4). Proteins to be degraded are polyubiquitylated by three enzymes: ubiquitin activating enzyme E1, ubiquitin conjugating enzyme E2, and ubiquitin ligating enzyme E3 ligases [66]. Most of the yeast enzymes that are involved in ubiquitination of a substrate protein are conserved in humans (Table 2 and Table 3). The first step in the UPS is ATP-dependent activation of ubiquitin by binding it to the E1 enzyme [67]. Ubiquitin is a protein of 76 amino acids having at least seven highly conserved lysine residues (K6, K11, K27, K29, K33, K48, and K63) from yeast to humans [61]. The activated ubiquitin is transferred to E2, which in conjunction with E3 enzyme transfers the ubiquitin molecule to the substrate proteins [68]. The ubiquitin conjugated to substrate protein can be further elongated with several ubiquitin forming polyubiquitinated proteins, which determines the fate of the protein inside a cell. In general, lysine residues are the ubiquitin binding sites in a substrate protein; however, serine, threonine, and cysteine have also been involved in ubiquitin conjugation. The binding of one ubiquitin molecule in a substrate protein is referred to as monoubiquitylation, while the binding of multiple ubiquitin molecules to a substrate is associated with its degradative fate. Meanwhile, recent studies have revealed that several monoubiquitylated proteins are also found to be degraded by the proteasome [69]. Ubiquitin can bind to a substrate protein at multiple lysine residues within the amino acid chain leading to multiubiquitination or it can elongate the existing monoubiquitinated sites making a chain of polyubiquitin. The polyubiquitylation of a substrate protein is the characteristic degradative signal, which is recognized by the proteasome [70].

The eukaryotic proteasome is a conserved multi-subunit proteolytic 26S complex composed of two subcomplexes: 20S core complex and 19S regulatory complex (Table 4). The abundance of characteristic polyubiquitinated proteins are positive regulators of proteasome assembly and formation [158]. The substrate entry into the core 20S complex is regulated by the regulatory 19S complex, which plays an important role in the recognition of the polyubiquitylated substrate protein, deubiquitylation of the substrate protein, unfolding the target protein, opening the gate of the core complex, and transporting of the protein to the core complex for degradation [159]. Several proteins work together to form a lid and a base of the regulatory complex. The core 20S complex is a barrel-shaped cylindrical structure formed by four stacks of heptameric rings of α (1–7) and β (1–7) subunits [160]. The rings formed by α-subunits are located on the outer side of the proteasome as a gate for the core complex. While the inner rings of heptameric β-subunits are the proteolytic site, β1, β2, and β5 have caspase-like, trypsin-like, and chymotrypsin-like proteolytic properties, respectively [161]. The proteasomal protein quality control is an essential process and requires high energy for the assembly of proteasomal subunits. The expression of the proteasomal subunits are tightly regulated by the transcription factor regulatory particle non-ATPase (Rpn4p in yeast), which recognizes a specific nonameric sequence (5′-GGTGGCAAA), also referred to as a proteasomal-associated control element (PACE), present in the promoter region of the majority of the genes encoding subunits of proteasome [162]. Importantly, it has been reported that nuclear translocation of Rpn4 has been found to be associated with activation of other transcription factors, including Hsf1p, yeast AP-1 (Yap1p), and pleotropic drug resistance (Pdr1p) [163].

### 2.3. Autophagy

Autophagy is the major process for turnover of cellular components including organelles in eukaryotes [196]. Autophagy is a process that delivers components of the cytoplasm and organelles to lysosomal compartments, depending upon the cellular survival requirements [197]. Various mechanisms of activation of autophagy exist for the sole purpose of adapting through cellular stresses. Autophagy is categorized into three groups: microautophagy, chaperone-mediated autophagy and macroautophagy [198]. Microautophagy is a process of degradation of cellular components by direct engulfment of the cytosolic components into lysosomes [199]. Chaperone-mediated autophagy is selective autophagy, where chaperones mediate the delivery of the specific cellular components or proteins that have KFERQ motifs to the lysosomal vesicles for degradation [200]. Most of the cellular components, including organelles and protein aggregates, are degraded through macroautophagy [201]. Macroautophagy is broadly categorized into selective and non-selective macroautophagy. Previously, it was thought that the autophagy is a non-selective process, but it is now becoming clear that selective autophagy also plays a significant role in targeted degradation of cytosolic components [202]. In fact, numerous specific autophagy receptors (SARs) have been identified in autophagic vesicles that help the delivery of the imbedded cargo to the lysosomal/vacuole compartment [203]. Non-selective autophagy is a bulk degradation process and regarded as the most essential process required for cellular component turnover. Regardless of the type of autophagy and its purpose in a eukaryotic cell, 19 core autophagy related (Atg) proteins have been identified to take part in formation of autophagic vesicles [204]. Interestingly, most of these core Atg proteins are conserved from yeast to humans (Table 5). In fact, it was primarily the yeast studies that have unraveled the majority of molecular processes involved in human autophagy.

#### 2.3.1. Core Autophagy Machinery in Yeast

**Figure 5:**
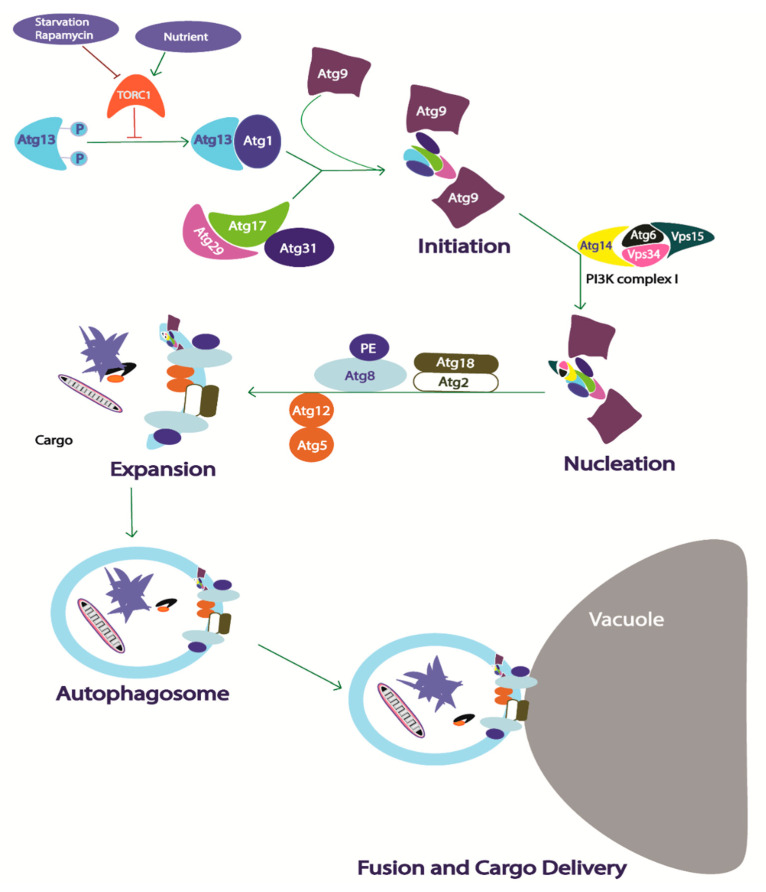
Schematic diagram of autophagosome formation and delivery of embedded cargo to the vacuole. Arrow, activates; Blunt arrow, blocks/inhibits; Atg, autophagy related; PI3K, phosphatidyl inositol 3 kinase; PE, phosphatidylethanolamine. Autophagosome formation is completed in four steps starting from the activation of Atg13 and formation of Atg1 kinase complex at the pre-phagosome assembly site. The second step involves the initiation of the phagophore formation by recruiting Atg9 protein. The phagophore nucleation occurs following Atg9 conjugation, where complexation with phosphatidyl inositol 3 kinase complex I occurs. The expansion of the double walled phagophore occurs embedding the cytosolic cargo that will ultimately give rise to autophagosome. The mature autophagosome then delivers the embedded cargo to the vacuole for degradation.

The core autophagy proteins can be categorized in five complex modules based on their function during autophagosome formation [204]. Autophagy has been characterized by distinct stages, starting from the phagophore assembly site (PAS), where it recruits several proteins to form double membrane phagophores, to the delivery of the embedded cargo to the lysosome/vacuole (Figure 5). The autophagy related 1 (Atg1) kinase complex involving five Atg proteins (Atg1, Atg13, Atg17, Atg29, and Atg31) assembles at PAS upon activation, initiating the autophagy [226]. During starvation or rapamycin treatment, the target of rapamycin complex 1 (TORC1) gets inactivated, and Atg13, a phosphorylation substrate of TORC1, gets dephosphorylated [241]. The dephosphorylated Atg13 conjugates with Atg17 and associates with Atg1, which helps to form the pentameric Atg1 complex (Atg1-Atg13-Atg17-Atg29-Atg31 complex) [242]. The Atg1 kinase complex recruits other proteins at the PAS, including Atg9, for initiation of the expansion of double membrane phagophore [243]. The expansion of the phagophore at this point requires phosphatidyl inositol-3 kinase (PI3K) complex I activity generating required phosphatidyl inositol-3 phosphate (PI3P). PI3P is essential for elongation of the phagophore by recruiting the Atg2-Atg18 ubiquitin-like conjugation complex, Atg8-lipidation complex, and Atg5-Atg12 conjugation complex [244]. In yeast, Atg8 has been found to be the most important autophagy-associated marker. Atg8 is produced in yeast as a precursor, which is processed by a cysteine protease Atg4 and ubiquitin-like modification system involving E1 enzyme like Atg7 and E2 enzyme-like Atg3, respectively [245]. This processing of Atg8 leads to the formation of the Atg8 lipidation complex comprising the Atg8 protein and phosphatidyl ethanolamine complex. Similarly, Atg12 also gets modified by two enzymes, E1 enzyme Atg7 and E2 enzyme Atg10, before conjugating with Atg5, forming the complex [246]. The phagophore expansion leads to engulfment of the autophagy cargo, including organelles, protein aggregates, lipids, and other unwanted cellular contents depending upon the cellular requirement and ultimately forms a double membrane autophagosomes [247]. The autophagosomes containing cellular cargo then get delivered to the lysosome in mammalian cells or the vacuole in yeast for degradation and recycling.

**Figure 6:**
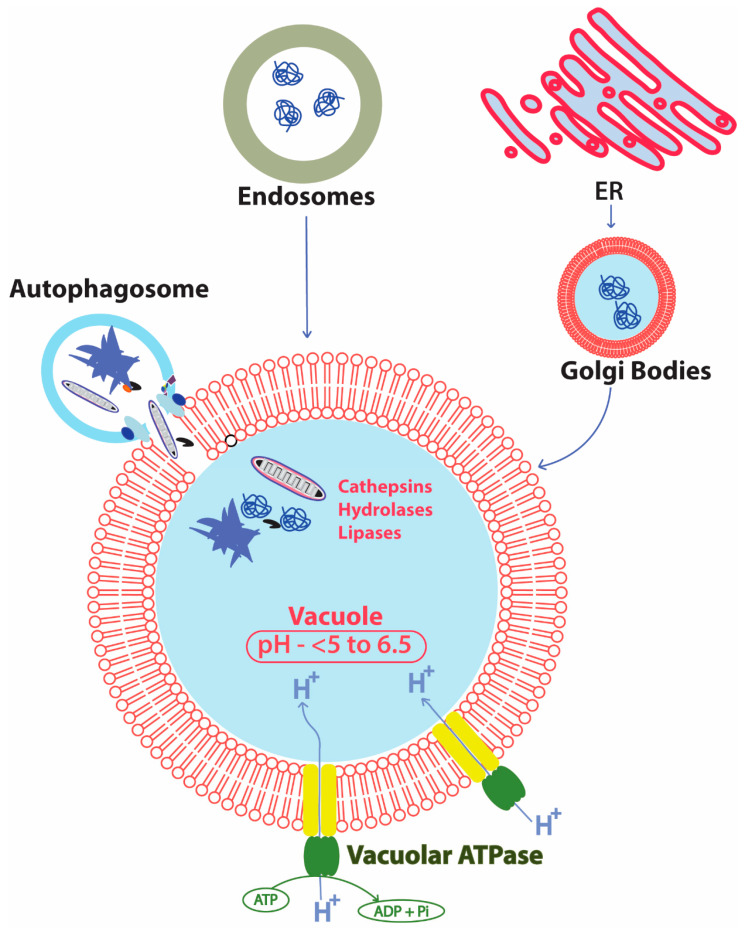
Different routes of delivery of cytosolic materials into the vacuole of yeast highlighting the fusion of autophagosomes with vacuole and energy-dependent role of V-ATPase in maintaining the pH of vacuoles. In the figure, the fusion of the autophagosome and the lysosomal membrane releases the autophagosomal cargo to the acidic lumen of the vacuole in yeast where these cargoes are degraded with the help of vacuole resident pH dependent cathepsins, hydrolases, and lipases. The pH of the vacuolar lumen is tightly regulated by an ATP dependent proton pump, the V-ATPase. Apart from the autophagosome mediated delivery of cargo inside vacuoles, endocytic vesicles and Golgi bodies carrying defective proteins are also delivered to the vacuole for degradation. Lysosomes may also degrade proteins and help protein turnover by direct engulfment of some cytosolic proteins, the process referred to as microautophagy.

The lysosome and vacuole provide similar acidic environments, conserved proteases, nucleases, lipases, and other hydrolytic enzymes for degradation of the autolysosomal cargo [248]. The delivery of unwanted cytosolic components into the vacuole generally takes place through multiple processes including autophagosomes (Figure 6) [249]. The fusion of the autophagosomes and lysosome/vacuole occurs with the help of tethering proteins mediating the binding of Atg8 in the autophagosome membrane and soluble N-ethylmaleimide-sensitive fusion attachment protein receptor (SNARE) complex present in lysosomal membrane [250]. The degradation of the lysosome/vacuole delivered cargo is dependent on the pH of the lysosome/vacuole. The acidic environment favors the activity of lysosome/vacuole resident enzymes, whereas alkalization leads to the impairment of clearance of cargo [251]. The pH inside the vacuole/lysosome is regulated by the vacuolar ATPase (V-ATPase) transmembrane proton pump present in the vacuole/lysosome, and also present on other intracellular organelles including the ER and Golgi complexes [252]. These proton pumps maintain the acidity of the organelle by pumping the protons in the luminal part in an energy dependent manner, while its impairment has been found to increase the pH of the organelle [253]. Ageing in yeast has been found to be a major contributor of vacuolar alkalization, which is found to be caused by defective assembly of V-ATPases in lysosomal membranes [210].

Interestingly, the components of the V-ATPase and regulation of its reversible assembly are also conserved in yeast (Table 6) [254]. The V-ATPase proton pump has been divided into two major subunits, namely V0 and V1 subunits. The V1 subunit is involved in hydrolysis of ATP and the V0 part is important for the proton pump activity, which maintains the pH of the intracellular organelles [252]. Both the subunits V0 and V1 comprise of multiple subunit proteins. The reversible assembly of the V0 and V1 has been found to be crucial for regulation of vacuole/lysosomal cargo degradation, which has been implicated in pH homeostasis and longevity [255].

#### 2.3.2. Conservation of Autophagy Regulation in Yeast Models

**Figure 7:**
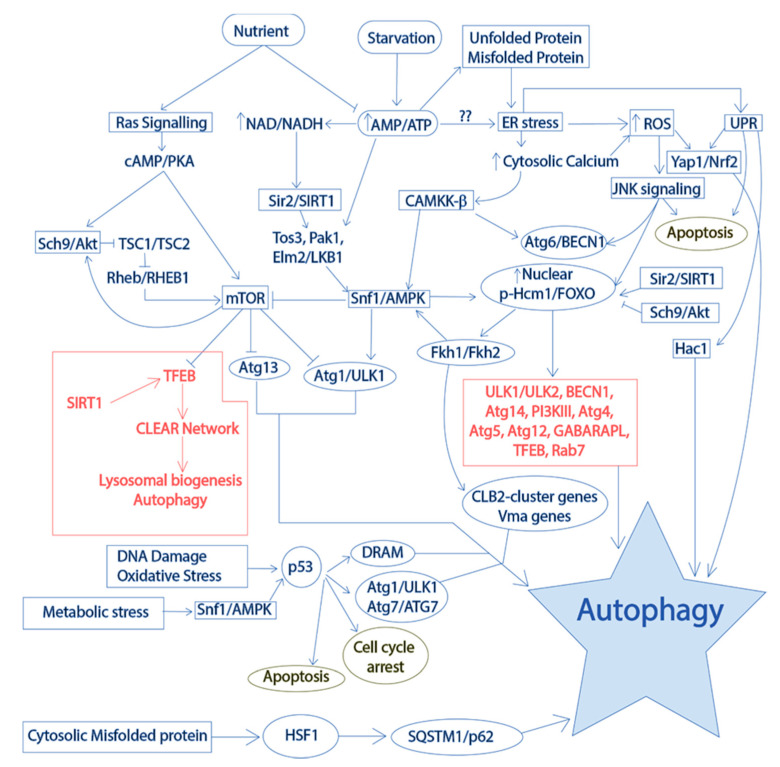
Some possible cell pathways that converge to yeast autophagy in response to various stimuli, including nutrient sensing, calcium homeostasis, oxidative stress, misfolded proteins, and UPR. Nutrient supplementation in a eukaryotic cell activates RAS signaling activating protein kinase activity leading to the activation of mTOR, whereas nutrient deficiency activates AMPK/Snf1 via sensing AMP/ATP ratio changes. Sir2/SIRT1 gets activated by increasing NAD^+^ in an energy-deficient environment and activates several downstream targets via deacetylation of its targets. Nutrient starvation may also have role in inefficient protein folding triggering the formation of unfolded proteins and misfolded proteins. The folding aberrations in ER are indicated by altered calcium levels in the cytosol and ER lumen. The increase in cytosolic calcium activates CAMKKβ, an upstream kinase of AMPK. The calcium abundance may also cause calcium overloading of mitochondria and enhance ROS formation. The presence of unfolded protein activates UPR, antioxidant response, MAPK signaling, and FOXO signaling and supports autophagy induction. However, chronic ER stress and JNK signaling may trigger apoptosis activation. The interaction between AMPK/Snf1, FOXO/Hcm1, and SIRT1/Sir2 induces autophagy and cytoprotective processes, whereas activation of mTOR, chronic ER stress, mitochondrial calcium overloading, and ROS may decrease survival possibly via inhibiting autophagy. During metabolic stress, DNA damage and oxidative damage, autophagy may also be activated via p53 activation. Meanwhile, p53 may also trigger apoptosis and cell cycle arrest depending on the cellular environment. Regulation of autophagy is a complex interaction of intracellular cues acting at several different levels. Some mechanisms depicted in the figure in the red boxes are not reported to be present in yeast.

In addition to the conservation of molecular machinery for protein turnover from yeast to humans, the mode of regulation is also conserved. Several molecular events, organelles, and external factors play important roles in regulating autophagy at different levels (Figure 7). The crosstalk between nutrient availability, mitochondria, protein folding mechanisms, and lysosomes is a major regulator of autophagy [276,277]. The protein folding occurs mainly in the ER and cytoplasm of a eukaryotic cell. In normal conditions, protein folding in the cytoplasm is carried out with the help of chaperones, especially heat shock proteins (HSPs) of the HSP70 family [29]. Any aberrations in protein folding will trigger a heat shock response through activation of heat shock factor 1 (HSF1), which enhances expression of chaperones for repairing the folding and promotes activation of autophagy for clearance of the unfolded or misfolded proteins [278].

Initially, it was found that the activation of autophagy initiates from starvation and it shuts off in nutrient rich conditions [279]. It has been found that sucrose non-fermenting 1 (Snf1)/adenosine monophosphate kinase (AMPK) becomes activated with the decrease in cellular energy or ATP to AMP ratio. The activated Snf1/AMPK phosphorylates ATG1 in yeast and Unc-51-like autophagy activating kinase (ULK1), triggering the initiation of autophagic phagophore formation [280]. The activation of AMPK requires activation of upstream kinases activity mediated by sirtuin (SIRT1/Sir2), the NAD^+^ dependent histone deacetylase [281,282]. SIRT1/Sir2 regulates activity of multiple substrates inside cell apart from histones. The activity of SIRT1/Sir2 is important for cell survival, life span, and longevity [283]. In addition, the energy depletion in a cell not only reduces the AMP/ATP ratio, but also converts more NADH to NAD^+^ to generate energy through the electron transport chain [284]. The cellular environment with a higher level of NAD^+^ activates SIRT1 expression and SIRT1-mediated deacetylation of its targets [285]. In mammals, another important target of SIRT1 is transcription factor EB (TFEB), the master regulator of lysosome biogenesis and autophagy [2]. In addition, DNA damage, metabolic stress, and oxidative damage of cells activates tumor suppressor protein p53 mediated activation of autophagy and cell cycle arrest [286]. It has been reported that activation of p53 during metabolic stress also requires AMPK mediated phosphorylation [287]. The p53-dependent cytoprotective function is dependent on its nuclear translocation, while cytosolic localization of the protein has been found to trigger apoptosis and cell death. The acetylation of p53 has been found to be associated with apoptosis activation [288]. Hence, Sir2/SIRT1 mediated deacetylation of p53 may have cytoprotective role as p53 is also a known substrate for Sir2/SIRT1 [289]. However, chronic cellular damage to the cell is the major reason for the apoptosis activation and cell death via p53 acetylation.

The mitochondrial electron transport chain is the primary source of intracellular reactive oxygen species [290]. Additionally, the mitochondria also serve as the major source of iron-sulfur clusters, heme, and phospholipids that are important for normal cell function. In relation to autophagy regulation, mouse models with defective mitochondria are shown to impair the lysosomal degradation [291]. The impairment of lysosome was attributed to the enlargement of lysosome associated membrane protein 1 (LAMP1)-positive lysosomal vesicles and the elevation of pH in the lysosome causing defective hydrolytic activity [292]. The mitochondrial dysfunction mediated acute impairment of lysosome triggers lysosomal regeneration or biogenesis through activation of TFEB [293]. The TFEB activation changes its subcellular localization from cytosol to nucleus and enhances its binding to the target genes. However, another transcription factor zinc finger with KRAB and SCAN domains 3 (ZKSCAN3) finely tunes expression of TFEB target genes by restricting TFEB-DNA binding by recognizing similar promoter regions [294]. In nutrient deficient conditions, ZKSCAN3 translocates to the cytoplasm and TFEB to nucleus. In chronic mitochondrial stress, the impairment of lysosomes is not replenished by lysosomal biogenesis. This could be due to irreversible cell damage caused by impairment of mitochondrial turnover or mitophagy.

On the other hand, the presence of nutrients in the cellular environment activates TOR complex (TORC), which prevents conjugation of Atg1p and Atg13p [241]. The nutrient availability has also been found to activate Ras signaling in both yeast and mammals [295,296,297]. In mammals, Ras signaling activates protein kinase B (Akt) in a cyclic AMP (cAMP)-dependent manner through adenylate cyclase activation [298]. The protein kinase B (Akt) is a serine/threonine kinase that activates TORC by deactivating tuberous sclerosis complex (TSC1 and TSC2), inhibitors of TORC [299]. Although yeast Ras signaling has not been greatly explored, the TSC complex and TORC in yeast are similar to that of mammals. The yeast TORC consists of two components TOR1 and TOR2 complex, which works in conjunction with several other proteins to have its autophagy inhibitory effect [300]. Nutrient abundance regulation of TORC plays a positive role in TOR activation, which inhibits autophagosome formation initiation. The TOR kinase activity phosphorylates several transcription factors that induce autophagy by enhancing expression of autophagy-related genes [301]. The TOR kinase activity has been reported to inhibit the Snf1/AMPK activation and downstream signaling [302]. The activated TOR can also phosphorylate TFEB rendering it inactive and limiting its nuclear translocation ultimately inhibiting autophagy and lysosomal biogenesis [303]. The AMPK inactivation and TOR activation subsequently downregulate autophagy, while its reversal induces autophagy. In addition, glucose abundance also increases the hexokinase II activity producing glucose-6-phosphate. The hexokinase II is also reported to inhibit TORC in glucose deficient condition, while glucose abundance spares TOR to get activated [304].

Another important cellular event that can activate autophagy is calcium ion turnover in the cytoplasm [305]. The endoplasmic reticulum harbors most of the calcium in a cell, where it plays a crucial role in energy-dependent oxidative protein folding and stability [306]. Furthermore, calcium is also present in other organelles, including lysosomes and mitochondria. The balance of the ER intraluminal concentrations of calcium depends on the energy requiring voltage-gated calcium channels [307]. Energy depletion or conditions leading to ineffective transport of calcium has an important role in protein folding and calcium homeostasis [308]. An increment in the cytoplasmic calcium concentration has been reported to increase oxidative damage [309]. Calcium overloading of mitochondria may have detrimental effects in mitochondrial function and may cause serious damage [310]. In the meantime, subtle increments in calcium levels may have important regulatory effects on autophagy activation [305]. Increased cytosolic calcium may indirectly inform protein folding aberrations. The depletion of energy in the cell may be a cause for such display, while the cytosolic calcium signals by activating Ca^2+^/calmodulin dependent protein kinase kinase β (CAMKKβ) pathway to activate autophagy to replenish the energy deficits [311]. Both the energy depletion and protein misfolding need to be rectified for proper cell function. The CAMKKβ senses increased calcium and gets phosphorylated, which in turn activates AMPK, beclin 1 (BECN1) and induces autophagy [312,313,314]. The calcium ions present in lysosome/vacuole are important for regulating the pH of the vesicle [315]. The increased leaching of calcium from lysosomal compartment into cytosol reduces the acidity, which in turn impairs the activity of lysosome resident enzymes.

Fork head (Fkh) transcription factors are one of the major classes of autophagy regulators in almost all eukaryotic systems [316]. Although nearly 18 subfamilies of Fkh family have been reported in mammalian system, only two subfamilies are found to be conserved in yeast [213,317]. Homologs of mammalian fork head box (FOX) transcription factors FOXO and FOXM, namely high-copy suppressor of calmodulin 1 (Hcm1) and Fkh1/Fkh2, are found in yeast. Hcm1 is the master regulator of Fkh1/Fkh2 expression, while its nuclear exclusion during ageing in yeast is reported to cause vacuolar alkalization [210]. The nuclear exclusion of Hcm1 and associated vacuolar alkalization impairs autophagic clearance in ageing cells resulting in loss of proteostasis. The Snf1/AMPK, a major regulator of autophagy in yeast, phosphorylates Hcm1, which enhances its nuclear translocation and causes subsequent expression of target genes [209]. The expression of transcription factors Fkh1/Fkh2 through Hcm1 increase expression of their target genes, including *Snf1,* in a positive feedback loop inducing autophagy in yeast [318]. In a mammalian system, nuclear translocation of FOXO transcription factors have been reported to activate ULK1/ULK2, Sestrin 3 (Sesn3), beclin 1 (BECN1), PI3K, Atg14, Atg4, Atg5, Atg12, Microtubule associated protein 1 light chain 3 beta (MAP1LC3B), GABA type A receptor associated protein like 1 (GABARAPL1), TFEB and Rab7—a member of the RAS oncogene family [319,320,321]. The nuclear translocation of FOXOs is also dependent on SIRT1-dependent deacetylation, indicating co-expression of FOXOs with SIRT1 as very crucial for cell survival [212,322]. How yeast FOXO homologs are involved in regulating autophagy remains elusive. In a recent study, Fkh1/Fkh2 function in collaboration with Sir2 was found to be cytoprotective, while reduced activity of these transcription factors reduced cell survival [212]. In the meantime, the Akt mediated phosphorylation of FOXOs can trigger its cytosolic translocation thus inhibiting expression of autophagy-related genes [323]. Apart from autophagy regulation, Fkh1/Fkh2 expression is also important in yeast for cell cycle progression, apoptosis, stress resistance, DNA damage repair, oxidative stress, and ageing [212].

The increase in oxidative stress during ageing is a major contributor of autophagy impairment [324]. During electron transport in the mitochondrial membrane, formation of superoxide free radicals is normal. The loss of electrons from the electron transport chain also contributes to generation of free radicals. These free radicals are generally neutralized by various oxidative stress response proteins [325]. The level of reactive oxygen species (ROS) and reactive nitrogen species (RNS) inside cells are delicate and are balanced in subtle manner. A slight increase in ROS and RNS may be indicative of important molecular changes inside cell. However, a higher rise in oxidative stress may signify cellular damage and the requirement for an oxidative stress response and antioxidants [326]. The enhanced oxidative stress activates oxidative stress response genes by activating transcription factors nuclear factor erythroid 2–related factor 2 (NRF2) in mammals and Yap1/suppressor of kre null (Skn7) in yeast [327]. The activated antioxidant transcription factors translocate into the nucleus, where they recognize specific sequences in the promoter region, also referred to as antioxidant response elements (ARE). The binding of the transcription factors to these unique regions of promoters enhances transcription of downstream phase I, II, and III detoxifying genes and autophagy related genes [328,329]. However, an abnormal oxidative environment may also be favorable for disulfide bond formation between thiol groups of cysteine residues of several proteins, which may also lead to protein aggregation inside cell [330]. Autophagy replenishes the oxidative damage via clearance of the damaged proteins, oxidized lipids, protein aggregates, and organelles. The mitogen activated protein kinase (MAPK) signaling-mediated autophagy becomes activated during oxidative stress [331]. In eukaryotes, MAPK kinase kinase (MAPKKK), MAPK kinase (MAPKK), and MAPK are sequentially phosphorylated for signal transduction via MAPK signaling [332]. Despite its crucial role in regulating cell survival and cell death via autophagy and apoptosis, MAPK signaling is demonstrated to be involved in pexophagy and mitophagy in yeast cells [224,333]. During increased oxidative stress in a cell, c-Jun N-terminal kinases (JNK) signaling, a type of MAPK signaling, gets highly activated which in turn activates FOXOs, BECN1 mediated autophagy, and c-Jun and c-Fos transcription factors mediate expression of autophagy-related genes [334,335,336]. However, prolonged chronic oxidative damage in a cell may trigger apoptosis through JNK signaling [337].

Lastly, the autophagic clearance of cellular garbage is also dependent on lysosomal (in mammals) and vacuolar (in yeast) degradation [338]. The degradation in lysosomes/vacuoles is dependent on the pH of the organelles, which is regulated by the intracellular membrane proton pump V-ATPase [339]. It is not only the proteins involved in the formation of V-ATPase complex that are conserved from yeast to humans, the mechanisms of assembly and disassembly of the subunits of V-ATPase in the organelles are also conserved [340]. Nutrient rich conditions are found to promote the V-ATPase assembly in both yeast and mammals, perhaps involved in reducing increased hydrogen ion concentration due to excessive glycolysis which in turn balances cytosolic pH due to higher metabolism [255,341]. The readily available energy required for V-ATPase activity during nutrient fed conditions could be another important player in the assembly of the proton pump. In yeast, the Ras/cAMP/PKA pathway has been reported to be involved in activation of assembly of V-ATPase subunits during nutrient fed conditions [342]. Additionally, regulator of ATPase of vacuoles and endosomes (RAVE) complex also regulates assembly of V-ATPase in vacuoles of yeast [343]. Recent studies suggest a nutrient rich environment supports the V-ATPase mediated TORC1 activation via Ras homolog enriched in brain (Rheb) protein. Rheb protein is activated by phosphorylated Akt/PKB in the vacuolar/lysosomal membrane and activates anabolic processes inside cells, including inhibition of autophagy and enhancement of protein translation [344]. The lysosomal/vacuolar membrane resident Ragulator-Rag complex is crucial in regulating the TORC1 activation [345]. Conversely, the optimal nutrient supply renders the V-ATPase subunits disassembled from the intracellular membranes, inhibiting the V-ATPase activity [346]. Interestingly, it was also found that the V-ATPase assembly is also enhanced during glucose and amino acid starvation. The glucose starvation mediated assembly of V-ATPase has been found to activate AMPK through axis inhibitor (AXIN)/liver kinase B1 (LKB1) pathway [347]. Such upregulation of V-ATPase assembly in the vacuolar/lysosomal membrane could help clear the cargo within autolysosomes and replenish the free amino acids and energy required for cell survival. Additionally, AMPK activation inhibits mTORC1 activity and supports catabolic processes by activating autophagy. In this way, regulated reversible V-ATPase assembly and dissociation regulates cellular homeostasis by controlling the major protein quality control mechanism including autophagy.

## 3. Proteostasis Failure in AD

AD is characterized by the accumulation of unwanted toxic protein aggregates including tau neurofibrillary tangles and Aβ aggregates (oligomers, fibrils, and senile plaques) [2]. From previous studies, it has been found that the protein quality control and the cellular defense system has failed severely during AD progression [348]. The impact of AD pathogenesis can be found at all the levels of the proteostasis network.

The increased phosphorylation of PERK and eIF2α, upregulation of CHOP signaling in temporal cortex, increased expression of BiP chaperones, HSP70 family proteins and increased splicing of XBP1 in temporal cortex and hippocampal tissue indicates increased UPR and chronic ER stress during AD pathology [349,350,351]. The activation of protein kinase activity of PERK has been demonstrated to activate Glycogen synthase kinase 3 beta (GSK3β), a major contributor of tau hyperphosphorylation in AD patients, in cell culture studies [352]. These evidences in support of chronic ER stress and UPR during AD provides rationale to explore therapeutic agents that can reduce ER stress or at least reduce protein synthesis in AD patients. Another important molecular marker of chronic ER stress is calcium dyshomeostasis, which is prevalent in AD [353]. The unacceptable calcium levels in the cytoplasm may lead to calcium overloading of organelles including mitochondria in neurons inhibiting respiratory growth. Impaired mitochondrial function and increased oxidative damage due to ROS formation during AD may partly be contributed by such abnormalities in neurons of AD patients [354]. Additionally, ER stress is one of the major causes of RNA-protein granules in eukaryotic cells. Meanwhile, recent studies suggest an association of tauopathies with the formation of such granules, hinting at an important role of impaired UPR in tauopathies implicated in AD [355]. The UPR inside a cell is generally backed up by UPS and autophagy.

Accumulation of high levels of ubiquitinated proteins during AD pathology strongly suggests impairment in UPS system [356]. The decreased proteasome activity in various parts of brains of AD patients signifies the impairment of UPS [357]. The oxidation of deubiquitinating enzymes (DUB)-mutated ubiquitin results in its aggregation or reduced expression of E1 and E2 enzymes in AD patients’ brains, which strongly implies its impairment during AD pathogenesis [358]. Under normal conditions, UPS degrades proteins that are soluble including soluble forms of Aβ and tau, while the hydrophobic forms of these proteins are degraded by autophagy [359]. Meanwhile, impaired UPS mediated degradation of soluble Aβ40, and microtubule associated protein tau may lead to their accumulation over time. With other molecular accomplices including activated GSK3β during AD progression, tau gets hyperphosphorylated and forms neurofibrillary tangles inside cells [360]. On the other hand, the presence of hydrophobic Aβ42 in the neurons may seed the aggregation of soluble Aβ40 [361]. In a normal cell, the impaired proteasome and ubiquitylated proteins can also be degraded through autophagy.

The impairment of autophagic clearance of aggregated proteins in AD brains may result in Aβ plaque formation followed by the exocytosis of amyloid aggregates or cell death [362]. The impaired UPS and autophagy defects also result in reduced clearance of tau tangles present in brains of AD patients, impairing the synapsis and cognition in patients [363]. Recent studies revealed important roles of UPS in regulating Atg9 availability, which in turn regulates autophagy progression [364]. In general, the nutrient status of a cell regulates UPS-dependent regulation of several cellular proteins and transcription factors that drive cell fate. During ageing, cells are deprived of nutrients due to their impaired nutrient sensing, which may have crucial roles in impairing the UPS system [365]. The impaired UPS in AD patients leads to the accumulation of ubiquitylated proteins, aggregation of misfolded proteins, and impairment of transcriptional regulation in ageing cells. The impaired crosstalk between the UPS and autophagy, resulting in progressive destruction of the cellular proteostasis network during ageing, seems to be the major culprit in AD pathology.

In normal brains, the neuronal cells are highly efficient in clearing the autophagosomes as compared to other tissues of the human body [366]. However, during ageing the efficiency of autophagic clearance gradually decreases, rendering the aged neurons prone to death [367]. The accumulation of very high amounts of autophagic vesicles in dystrophic neurites of AD patients clearly supports impairment of autophagic clearance [368]. Interestingly, the various stages of autophagic vesicles present in numerous defective neurons are filled with Aβ peptides, APP, and β-C-terminal fragment (βCTF) of the APP [362]. It has been clear that the autophagosomes are the primary site for Aβ generation by γ-secretase mediated cleavage of the βCTF [369]. The formation of Aβ peptides in normal brain is accompanied by enhanced clearance by the cathepsin-mediated degradation, while AD brains are ineffective in such clearance [370]. Genetic variations in cathepsin D have been found associated with higher accumulation of Aβ in AD patients [371]. One reason behind such observations is the alkalization of the lysosomes due to impaired V-ATPase assembly [253]. In recent years, modulation of reversible V-ATPase assembly has been a cherished topic in AD therapeutics. The soluble forms of intracellular Aβ are likely to be diffused in cytosol and are degraded by the help of heat shock factor activation and subsequent expression of heat shock proteins [372]. However, reduced expression of heat shock proteins in AD pathology has been observed, suggesting one possible mechanism of impairment of protein misfolding, clearance, and accumulation of Aβ peptides in dystrophic neurites [372,373]. The hydrophobic forms of Aβ (Aβ42) may still remain in acidic lysosomes, where the formation of oligomers and fibrils are favored [374]. In addition, as mentioned previously, familial AD has been found associated with mutations in several genes including PSEN1, the catalytic subunit of γ-secretase found in endosomes, autolysosomes, and autophagosomes [375]. The PSEN1 protein has also been found to regulate the autophagosome lysosome fusion by modulating telencephalin (TLN) clearance [376,377]. The mutated version of the PSEN1 are found ineffective in regulating telencephalin levels, resulting in ineffective fusion of the autophagosomes and lysosomes, contributing to another reason for accumulation of autophagic vesicles during AD. Apart from these, PSEN1 is involved in the calcium balance, apoptosis, synaptic plasticity, and proteolysis of several proteins, including APP and cell adhesion through its catalytic activity. In addition, γ-secretase independent activity of PSEN1 has been shown to have a role in vacuolar acidification and UPR. The mutated PSEN1 in AD might have crucial role in impairing the vacuolar protease activity by modulating the vacuolar pH [362]. Additionally, the mutation in PSEN1 has been reported to reduce the expression of BIP chaperones, leading to enhanced ER stress [378].

The mitochondria are reserves of iron and during mitophagy release of oxidized iron (Fe^2+^) increases oxidative stress mediated by the Fenton reaction [379]. The release of oxidized iron can also occur during degradation of other iron rich molecules or proteins like ferritin. The interaction of oxidized lipids, proteins, and metals in lysosome with the free radicals may result in oxidation of the reacting molecules and their aggregation, ultimately malfunctioning of the lysosome [380]. It may result in production of auto-fluorescent ceroid particles referred to as lipofuscin, a hallmark of ageing [381]. Furthermore, excessive mitophagy due to chronic mitochondrial stress during AD may contribute to an increase in lipofuscin formation and increase oxidative damage, impairing the lysosomal degradation. In normal cells, the lipofuscins are neither degraded nor exocytosed, but can be diluted out by cell division [381]. However, terminally-differentiated neurons are unable to divide further, thus leading to accumulation of such particles in neuronal cells during ageing [382]. The lipofuscin has been reported to interact with autophagosomes, lysosomes, and proteasomes inside cells and impair the cellular protein homeostasis implicated during AD progression [383,384]. Additionally, iron overloading is a common feature of AD brains and is accompanied by ferroptosis-mediated cell death. Recent evidence supports the high association of autophagy-related genes in increasing the ferroptosis-mediated cell death [385]. In AD patients, events including monoamine oxidase hyperactivation, calcium dyshomeostasis, increased oxidative stress, lower expression of SIRT1, hyperactivation of CAMKKβ and MAPK signaling, defective assembly of V-ATPase, alkalization of lysosomes, defective autophagosome-lysosome fusion, and other important molecular players may render the neurons to nurture accumulation of autophagic vesicles [253,354,362,386,387,388,389,390].

In summary, the impairment of most of the mechanisms of protein synthesis, folding and quality control governs the accumulation of protein aggregates in AD patients’ brain. Hence, it is of utmost importance to study the proteostasis network to understand the ways to modulate the balance of protein turnover and recover overall cellular function. This will aid the design of potential therapeutic agents.

## 4. Future Perspectives of Using Yeast as a Model Organism for Alzheimer’s Disease

The power of using yeast as a model organism to study AD comes from the underlying similarity to human cells, including neurons, ease of using yeast to understand molecular processes, and the availability of analytical platforms [15,24]. The intriguing fact that most of the processes of ageing are also conserved in yeast makes it an appropriate model for age-related neurodegenerative diseases. *S. cerevisiae* cells bud asymmetrically for up to 20–25 cycles, indicating a limited replicative lifespan [16]. The division of cells produces smaller daughter cells and leaves scars on the cell surface [391]. The ability to readily track these scarred cells provides a convenient platform to study young cells and aged cells [392]. In addition, replicative ageing of a yeast cell also increases the size of the yeast, making it possible to explore yeast using size–based, cutting edge technological platforms such as single cell analyzers [393,394]. The development of yeast models that expresses toxic proteins of AD, including the APP, Aβ, and tau protein in the past, has provided increasing interest among researchers [17,19,25,395,396]. In addition, knowledge gained from the use of unmodified yeast continues to provide answers to research questions pertaining to fundamental aspects of eukaryotic cells, which could be crucial in disease pathology. The outcomes observed in yeast after heterologous expression of such human proteins has served as the basis for further studies and unravelling of the molecular pathogenesis caused by these proteins. These yeast models have provided unique frontier for screening of both cytoprotective and cytotoxic compounds [18,21,397,398,399]. Several yeast reporter systems have also been developed, which aid AD research and provide directions for future research [400,401].

The conservation of genetics, protein synthesis, processing, and quality control system of eukaryotes including the ER stress, formation of stress granules, chaperones, heat shock stress response, unfolded protein response, ubiquitin proteasome system, and autophagy advocates yeast as appropriate model for studies of disease involving proteostasis failure including AD. The data coming from yeast are consistent with observations in AD patients, and hence, can be utilized for further studies. Future studies involving complex interplay between the parts of proteostasis network can be easily accomplished in yeast models of AD, which have potential to provide clarity in AD pathogenesis.

The conservation of most of the core machinery of UPS and presence of homologs of most of the counterparts of the human UPS in yeast system offers promise for the future of drug discovery studies. Additionally, most of the core autophagy related proteins and proteins involved in lysosomal degradation are also conserved in yeast. In fact, it is the yeast system that has provided enormous amount of information on these fundamental processes of a eukaryotic system. Recent studies showing high involvement of ferroptosis and iron overloading in AD and its association with autophagy provide important opportunities that can be addressed with yeast studies, as some of the markers of ferroptosis and autophagy-related genes are conserved in yeast. Apart from these benefits for using yeast as a model, the ability of yeast to grow with defective mitochondria provides unique platform for studies involving mitochondrial dysfunction in yeast, which is crucial for UPS, autophagy, and lysosomal functioning. In such a scenario where mitochondria are defective, yeast can be very useful for finding compounds that can rescue and enhance mitochondrial inhibition or help mitochondrial regeneration, both types of study crucial for AD research. In contrast, such studies cannot be performed in any mammalian system, as mitochondrial respiration is crucial for cell survival in mammals.

Nevertheless, differences still exist due to the complexity of higher multicellular eukaryotes as compared to the unicellular simple eukaryotic yeast. However, heterologous expression of uncommon human proteins in yeast may still interact with some conserved proteins inside yeast that could still be beneficial for understanding its role in cell and finding the interaction network that will help to predict the future directions. In fact, the reduced complexity in yeast cell models and the possibility of reconstitution of human proteins in controlled environment makes yeast a fascinating tool to explore role of human proteins in disease pathology. At the same time, the lack of conservation of some mechanisms including immune system, cell to cell communications, and nervous system in yeast limits its use in understanding complex processes occurring in human tissues, while the conservation of most of the cellular signaling and processes still provide rationale for using yeast as a single cell model organism to study protein homeostasis network. Considering the high association of proteostasis network and AD progression, yeast will prove to be an important tool for finding therapeutics and understanding the molecular processes of AD and other similar diseases.

## Figures and Tables

**Table 1:** Conserved proteins of yeast unfolded protein response and their human homologs.

Yeast Protein	Human Homolog	Function	References
Kar2p/Bip/Grp78p	HSPA5/BiP/GRP78	Chaperone regulating activation of Ire1p	[42,57]
Ire1p	IRE1α IRE1β	Stress sensing and endonuclease activity	[58]
	PERK	Stress sensing and protein kinase	[59]
Hac1p	XBP1p	UPRE binding and expression of target genes	[59]
Sui2p	eIF2α	Eukaryotic translation initiation factor involved in protein translation regulation	[60]
Ypt1p	RAB1Ap	Regulates UPR by *HAC1* mRNA decay	[44]

**Table 2:** Yeast ubiquitins, E1 activating enzymes, and E2 conjugating enzymes conserved in humans that are involved in polyubiquitylation of substrate protein.

Yeast Protein	Human Homolog	Functions of Proteins in Yeast	Ref
**Ubiquitin**			
Ubi1, Ubi2, Ubi3, Ubi4	UBB, UBC	Cellular stress response and ubiquitination of proteins	[71]
**Ubiquitin activating E1 enzyme**			
Uba1	UBA1	Ubiquitin-mediated protein degradation	[72]
**Ubiquitin conjugating E2 enzyme**			
Ubc1	UBE2K	Major E2 enzyme works together with Ubc4, degradation of short-lived and aberrant proteins, increases protein during DNA stress, cellular stress response, vesicle biogenesis, and ERAD	[73,74,75]
Ubc2	UBE2A, UBE2B	K63 ubiquitination mediated oxidative stress response; turnover of Rpn4; DNA repair	[76,77]
Ubc3	UB2R1, UB2R2	Regulation of cell cycle progression; protein abundance during DNA stress	[78,79]
Ubc4, Ubc5	UB2D1, UB2D2, UB2D3, UB2D4	Major E2 enzyme works together with Ubc1; degradation of abnormal calmodulin and H3 histone; regulates levels of DNA polymerase	[80,81,82]
Ubc6	UB2J2	ERAD; ER membrane (Cytosolic) resident E2 enzyme	[83]
Ubc7	UB2G1, UB2G2	ERAD; inner nuclear membrane associated degradation; chromatin assembly	[84]
Ubc8	UBE2H	Regulates gluconeogenesis	[85]
Ubc9	UBC9	Anaphase promoting complex cyclosome mediated proteolysis; Cell cycle progression	[86]
Ubc10	UB2D1, UB2D2	Peroxisome biogenesis	[87]
Ubc13	UBE2N	DNA damage repair, DNA stress response	[88]

**Table 3:** Yeast ubiquitin E3 ligating enzymes conserved in humans.

Yeast Protein	Human Homolog	Functions of Proteins in Yeast	Ref
Ubiquitin ligase E3 enzyme			
Hul4	HECTD2	HECT ubiquitin ligase	[89]
Hul5	UBE3C	HECT ubiquitin ligase; essential for elongation of polyubiquitin chains in the misfolded proteins during heat shock response; ERAD	[90,91,92]
Rsp5	NEDD4L	NEDD4 family E3 ubiquitin ligase; regulates heat shock response; endocytosis and vesicle trafficking.	[92,93,94]
Tom1	HUWE1	HECT ubiquitin ligase; export of mRNA to cytosol; balancing histones; cell cycle regulation.	[81,95,96]
Ufd4	TRIP12	E3 ubiquitin ligase; involved in DNA stress.	[79]
Asr1	RNF165	Ubiquitin E3 ligase; regulates RNA Polymerase II subunits; actin cytoskeleton organization	[97,98]
Bre1	RNF40	E3 ubiquitin ligase conjugates with Rad6p and regulates histone methylation; oxidative stress response mediated proteolysis; double stranded break repair; pre-mRNA splicing.	[76,99,100,101]
Dma1, Dma2	RNF8	E3 ubiquitin ligase; regulates level of eIF2	[102]
Ssm4/Doa10	MARCHF6	RING-CH domain E3 ligase associated with ER and nuclear membrane; involved in ERAD,	[84]
Hel2	ZNF598	RING finger E3 ubiquitin ligase; excess histone clearance; involved in ribosome associated quality control	[103,104]
Hrd1	SYVN1	H2 ring finger E3 ubiquitin ligase; involved in UPR and ERAD	[105]
Fyv10	MAEA	Ring finger ubiquitin ligase; inhibits gluconeogenesis; anti-apoptotic; part of glucose-induced degradation-deficient (GID) complex	[106,107,108]
Irc20	SHPRH	Ring finger E3 ubiquitin ligase; involved in non-homologous end joining DNA repair pathway	[109]
Nam7	UPF1	RING-related E3 ubiquitin ligase; nonsense mutation mediated mRNA decay	[79]
Mot2	CNOT4	Ubiquitin ligase of CCR4-NOT complex; regulates transcription, post transcriptional modifications and mRNA degradation	[110,111]
Pep5	VPS11	Histone E3 ligase that enhances degradation of excess histones; vacuole biogenesis.	[103,112]
Pex2	PEX2	RING finger peroxisomal E3 ubiquitin ligase	[113]
Pex10	PEX10	Peroxisomal membrane associated E3 ubiquitin ligase	[114]
Pex12	PEX12	RING finger peroxisomal E3 ubiquitin ligase	[115]
Pib1	FYCO1	RING finger ubiquitin ligase of endosome and vacuole membranes	[116]
Psh1	TRIM25	E3 ubiquitin ligase for degradation of centromere binding protein	[117]
Rad5	HLTF	Ubiquitin ligase or DNA helicase involved in DNA damage stress response	[118,119]
Rad18	RAD18	E3 ubiquitin ligase complexes with PCNA; involved in post replication repair of DNA	[120,121]
Rmd5	RMND5A	RING finger E3 ubiquitin ligase; inhibits gluconeogenesis; part of GID complex	[106]
Rkr1	LTN1	RING domain E3 ubiquitin ligase; part of ribosome quality control complex; clearance of defective mRNA	[122,123,124]
San1	RNF13	E3 ubiquitin ligase; involved in selective clearance of defective aggregate prone proteins	[125,126]
Slx8	RNF10	RING domain ubiquitin ligase; required for genomic integrity	[127]
Snt2	PHF14	RING finger E3 ubiquitin ligase; oxidative stress response; degradation of excess histones.	[103,128]
Ubr2	UBR2	Cytoplasmic E3 ubiquitin ligase	[129]
Prp19	PRPF19	E3 ubiquitin ligase; DNA damage response; pre-mRNA splicing	[130,131]
Ufd2	UBE4B	Polyubiquitylation of substrate protein; stress response; ERAD of misfolded proteins	[132,133]
Hel1	ARIH1	RING finger E3 ubiquitin ligase; clearance of excess histones	[103]
Itt1	RNF14	E3 ubiquitin ligase; modulates termination of translation	[134]
Cdc53	CUL1	Cullin family E3 ubiquitin ligase; cell cycle progression	[135,136]
Cul3	CUL3	Cullin family E3 ubiquitin ligase; degradation of RNA Polymerase II	[137]
Rtt101	CUL4	Cullin subunit of a E3 ubiquitin ligase complex; cell cycle progression; double stranded break repair; rRNA decay	[138,139]
Skp1	SKP1	Major component of SCF (Skp1-Cullin-F-box) E3 ubiquitin ligase complex; V-ATPase assembly; cell cycle progression; DNA replication stress response	[79,140,141]
Elc1	ELOC	E3 ubiquitin ligase; genomic repair; degradation of RNA polymerase II	[137,142]
Hrt1	RBX1	Part of various ubiquitin ligase SCF complex	[143]
Cdc4	FBXW7	F-box protein; part of SCF-Cdc4 ubiquitin ligase complex; ubiquitination of cyclin-dependent kinases	[144,145]
Met30	FBXW7	Essential protein; F-box protein; part of SCF complex; heavy metal stress response; Cell cycle	[146,147]
Ela1	ELOA	F-box protein heterodimerizes with Elc1; subunit of Elongin-Cullin-Socs ubiquitin ligase complex	[137,148,149]
Hrt3	FBX9	F-box protein of SCF ubiquitin ligase complex	[150]
Rav1	DMXL1	Part of RAVE complex; V-ATPase assembly; role in endocytosis	[151,152]
Saf1	HERC2	F-box protein involved in cell quiescence	[153]
Cdc27	CDC27	Part of anaphase promoting complex (APC)/cyclosome, a ubiquitin ligase complex involved in the degradation of anaphase inhibitors	[154]
Cdc16	CDC16	Part of APC/C, a ubiquitin ligase complex involved in the degradation of anaphase inhibitors	[155]
Cdc23	CDC23	Part of APC/C, a ubiquitin ligase complex involved in the degradation of anaphase inhibitors	[155]
Doc1/Apc10	ANAPC10	Subunit of APC; Substrate ubiquitination	[156,157]

**Table 4:** Proteins of the yeast proteasome conserved in humans and their functions in yeast.

Yeast Protein	Human Homolog	Functions of Yeast Proteins	Ref
**Deubiquitinating enzymes (DUBs)**			
Ubp1	USP19	Ubiquitin specific protease—carboxy terminal hydrolase activity	[164]
Ubp2	USP28	Ubiquitin specific protease—deubiquitinates proteins, controls K63 during oxidative stress	[76]
Ubp3	USP10	Regulate transport between ER and Golgi; regulates osmotic stress response; deubiquitinates COPI and COPII proteins; increases protein during DNA stress	[165,166,167]
Ubp4/Ubp5	USP8	Deubiquitinates proteins inside endosomes that are bound to be delivered to vacuole for degradation; increases intracellular free ubiquitin.	[168,169]
Ubp6	USP14	Negatively regulates branched polyubiquitinated proteins from proteasomal degradation	[170]
Ubp7/Ubp11	USP21	Deubiquitinating proteins involved in cell cycle progression	[171]
Ubp8	USP22	SAGA-mediated histone 2B ubiquitination	[172]
Ubp9/Ubp13	USP46	Deubiquitinating ubiquitin-protein fusions	[129]
Ubp10	USP20	Ribosome biogenesis and rRNA formation; deubiquitinates several cellular proteins.	[173]
Ubp12	USP15	Deubiquitinates proteins	[174]
Ubp14	USP5	Regulates gluconeogenesis	[106]
Ubp15	USP7	Peroxisome biogenesis and cell cycle progression	[175,176]
Ubp16	USP16	Mitochondria associated deubiquitinating enzyme	[177]
Rpn11	PSMD14	Metalloprotease present in 26S proteasome; helps in deubiquitylation and proteolytic cleavage of substrates	[178,179]
Otu1	YOD1	Deubiquitylation of proteins ubiquitinated by Ufd2	[180]
Otu2	OTUD6B	Increases abundance of protein during DNA stress	[79]
Yuh1	UCHL3	Hydrolyses C-terminal of ubiquitin peptide bonds and release monomeric ubiquitin	[181]
**Proteasomal Core (20S subunit)**			
**α-subunit (** **α1-7)**			
Scl1	PSMA6	Essential for survival, alpha-1 subunit of 20S core machinery, degradation of protein substrates	[182]
Pre8	PSMA2	Alpha-2 subunit of 20S proteasome	[183]
Pre9	PSMA4	Alpha-3 subunit of 20S proteasome	[182]
Pre6	PSMA7	Alpha-4 subunit of 20S proteasome	[183]
Pup2	PSMA5	Alpha-5 subunit of 20S proteasome	[184]
Pre5	PSMA1	Alpha-6 subunit of 20S proteasome	[183]
Pre10	PSMA3	Alpha-7 subunit of 20S proteasome	[79]
**β-subunit (** **β1-7)**			
Pre3	PSMB6	Beta-1 subunit of 20S proteasome	[182]
Pup1	PSMB7	Beta-2 subunit of 20S proteasome	[185]
Pup3	PSMB3	Beta-3 subunit of 20S proteasome	[186]
Pre1	PSMB2	Beta-4 subunit of 20S proteasome	[182]
Pre2	PSMB5	Beta-5 subunit of 20S proteasome	[182]
Pre7	PSMB1	Beta-6 subunit of 20S proteasome	[182]
Pre4	PSMB4	Beta-7 subunit of 20S proteasome	[185]
**Regulatory Particle (19S subunit)**			
**RP base**			
Rpt1	PSMC2	ATPase of 19S RP	[187]
Rpt2	PSMC1	ATPase of 19S RP	[188]
Rpt3	PSMC4	ATPase of 19S RP	[188]
Rpt4	PSMC6	ATPase of 19S RP; spindle pole duplication; ERAD	[188,189]
Rpt5	PSMC3	ATPase of 19S RP	[190]
Rpt6	PSMC5	ATPase of 19S RP	[79]
Rpn1	PSMD2	Protein–protein interaction; proteasomal ligand recognition and non-ATPase subunit of 19S RP	[191]
Rpn2	PSMD1	Non-ATPase subunit of 19S RP	[79]
Rpn13	ADRM1	Ubiquitin receptor of proteasome	[192]
Rpn10	PSMD4	Assembly of regulatory particle; non-ATPase subunit of 19S RP	[193]
**RP Lid**			
Rpn3	PSMD3	Essential non-ATPase subunit of 19S RP lid	[194]
Rpn5	PSMD12	Essential non-ATPase subunit of 19S RP lid	[188]
Rpn6	PSMD11	Essential non-ATPase subunit of 19S RP lid; assembly and activity of proteasome	[195]
Rpn7	PSMD6	Essential non-ATPase subunit of 19S RP lid	[188]
Rpn8	PSMD7	Essential non-ATPase subunit of 19S RP lid	[188]
Rpn11	PSMD14	Essential non-ATPase subunit of 19S RP lid, degradation and deubiquitylation of substrate proteins, mitochondrial and peroxisomal fission.	[178]
Rpn12	PSMD8	Essential non-ATPase subunit of 19S RP lid	[61]

**Table 5:** Conservation of core autophagy related proteins and autophagy regulators from yeast to humans.

Proteins Involved in Yeast Autophagy	Human Homologs	Function	Ref
Sir2	SIRT1	Sirtuin family NAD^+^-dependent histone deacetylase that activates upstream kinases that activate Snf1/AMPK and activates TFEB and autophagy in mammalian system.	[205,206]
Tos3, Sak1, Elm1	LKB1, CAMKK	Upstream protein kinases that activates AMPK/Snf1	[207,208]
Hcm1	FOXO3	Expression of other fork head box transcription factor proteins and expression of various proteins involved in autophagy; nuclear localization and expression of target genes essential for vacuolar acidification; mitochondrial biogenesis and stress resistance	[209,210,211]
Fkh1, Fkh2	FOXM	Expression required for stress resistance, autophagy, and cell cycle progression	[212,213]
Sch9	AKT/PKB	Regulates autophagy negatively works together with TORC1	[214,215]
Tsc1, Tsc2	TSC1, TSC2	Inhibits TORC1 activity and activates autophagy	[216]
Rhb1	Rheb	Promotes TORC1 activity and inhibits autophagy	[217]
Snf1	AMPK	Activates Atg1; nutrient sensing; inactivates TORC1 complex and positively regulate autophagy	[208,218]
Torc1	TORC1	Blocks interaction of Atg1 and Atg13; nutrient sensing, negatively regulates autophagy	[219]
Gtr1	RAGA, RAGB	Part of Rag GTPase complex; regulates TORC1 activity	[220]
Gtr2	RAGC, RAGD	Part of Rag GTPase complex; regulates TORC1 activity	[220]
Ssk2, Ssk22	MAP3K4/MEKK4	MAPK kinase kinase for Hog1	[221]
Pbs2	MAP2K2/MEK2	MAPK kinase for Hog1	[222]
Hog1	MAPK14/p38	Involved in mitophagy and pexophagy	[223,224]
Slg1/Wsc1	WSCD2	Important for mitophagy; stress response	[224,225]
Pkc1	PRKCA, PRKCB, PRKCD, PRKCE, PRKCG, PRKCH, PRKCI, PRKCQ	Activation of Bck2 in turn activating Slt2 involved in pexophagy and mitophagy	[224]
Slt2	MAPK7/ERK5	MAPK involved in pexophagy and mitophagy	[224]
Atg1	ULK1, ULK2	Serine/threonine protein kinase activity; conjugates with Atg13; autophagy initiation regulation	[226]
Atg2	ATG2A, ATG2B	Conjugates with Atg18; forms Atg2-Atg18 complex; and involved in autophagosome expansion	[227]
Atg3	ATG3	E2-like enzyme for formation of Atg8 lipidation complex	[228]
Atg4	ATG4A, ATG4B, ATG4C, ATG4D	Cysteine protease that processes the inactive Atg-8 at carboxy terminus to give mature Atg8	[229]
Atg5	ATG5	Conjugates with Atg12	[230]
Atg6/Vps30	BECN1	Important part of PI3K complexes	[231]
Atg7	ATG7	E1 like enzyme involved in both Atg12-Atg5 and Atg8-PE complex formation	[232]
Atg8	LC3A, LC3B, LC3B2, LC3C, GABARAP, GATE16	Conjugates with PE to form lipidation complex; important part of autophagosome membrane; interacts with SNARE proteins to deliver the autophagosomal cargo; determines the size of autophagosome	[233]
Atg9	ATG9	The only transmembrane protein of autophagosome required for phagophore expansion; interacts with Atg2 and regulates autophagy	[234]
Atg10	ATG10	E2-like enzyme conjugating Atg5-Atg12	[235]
Atg12	ATG12	Forms Atg5-Atg12 complex involved in phagophore expansion	[230]
Atg13	ATG13	Activated by dephosphorylation during starvation, conjugates with Atg1 to initiate formation of phagophore assembly site	[236]
Atg14	ATG14	PI3K complex I component	[237]
Atg17	FIP200/RB1CC1	Atg1 kinase complex component involved in initiation of autophagy	[236]
Atg18	WIPI1, WIPI2	Conjugates with Atg2; forms Atg2-Atg18 complex; binds with PI3P; and involved in elongation of phagophore.	[238]
Vps15	PIK3R4/VPS15/p150	Protein kinase required for activation of Vps34	[239]
Vps34	PIK3C3/VPS34	PI3K catalytic component	[240]

**Table 6:** Proteins of the V-ATPase proton pump conserved from yeast to humans and their function in yeast.

Vacuolar ATPase Subunits	Yeast Proteins	Human Homologs	Function of the Yeast Protein	Ref
**V1 Subunit**				
A	Vma1	ATP6V1A	Site specific endonuclease activity; methionine restriction regulation of life span; stress response	[256,257]
B	Vma2	ATP6V1B1, ATP6V1B2	Proton pump of endomembrane; protein abundance during DNA replication stress	[79,258]
C	Vma5	ATP6V1C1, ATP6V1C2	Part of proton pump; required for assembly of V-ATPase subunits at vacuolar membrane	[259]
D	Vma8	ATP6V1D	Role in proton pumping and ATP hydrolysis	[260,261]
E	Vma4	ATP6V1E1, ATP6V1E2	Part of V-ATPase; protein content increases during DNA replication stress	[79,262]
F	Vma7	ATP6V1F	Part of proton pump; required for V-ATPase subunits assembly at vacuolar membrane	[258,263]
G	Vma10	ATP6V1G1, ATP6V1G2, ATP6V1G3	Part of proton pump and role in vacuole acidification	[264]
H	Vma13	ATP6V1H	Part of proton pump; activates and stabilizes v-ATPase	[258,265]
**V0 Subunit**				
a	Vph1, Stv1	ATP6V0A1, ATP6V0A2, ATP6V0A3, ATP6V0A4	Part of V-ATPase complex; regulates V-ATPase activity; present in vacuoles, Golgi bodies and endosomes	[266,267,268]
c	Vma3	ATP6V0C	Proteolipid subunit; Vacuole acidification; Copper and Iron homeostasis	[269,270]
c’	Vma11	ATP6V0B, ATP6V0C	Integral hydrophobic membrane proteolipid; required for proton pump	[271,272]
c”	Vma16	ATP6V0B, ATP6V0C	Part of V-ATPase	[271]
d	Vma6	ATP6V0D1, ATP6V0D2	Part of proton pump; required for V1 assembly at vacuole membrane	[273,274]
e	Vma9	-	V0 biogenesis; vacuole acidification; part of V0 subunit.	[275]

## Abbreviations

AD	Alzheimer’s Disease
Aβ	Amyloid beta
EOAD	Early Onset Alzheimer’s Disease
LOAD	Late Onset Alzheimer’s Disease
APP	Amyloid precursor protein
PSEN	Presenilin
GWAS	Genome Wide Association Studies
ER	Endoplasmic reticulum
UPR	Unfolded protein response
eIF2α	Eukaryotic translational initiation factor 2 alpha
mRNA	Messenger ribonucleic acid
Ire1	Inositol requiring element 1
Bip	Binding immunoglobulin protein
ATF	Activating transcription factor
CREB	cAMP response element binding
HAC1	Homologous to Atf/CREB1
Ypt1	Yeast protein two 1
Rlg	tRNA ligase
bZIP	Basic leucine zipper
UPRE	Unfolded protein response element
ERAD	Endoplasmic reticulum associated degradation
PERK	PKR like ER kinase
XBP1	X-box binding protein 1
SP1	Specificity protein 1
SP2	Specificity protein 2
CHOP	C/EBP homologous protein
GADD34	Growth arrest and DNA Damage 34
UPS	Ubiquitin proteasome system
ATP	Adenosine triphosphate
PACE	Proteasomal associated control element
Rpn	Regulatory particle non-ATPase
Yap1	Yeast AP-1
Pdr1	Pleotropic drug resistance 1
SAR	Specific autophagy receptor
Atg	Autophagy related
PAS	Phagophore assembly site
TORC1	Target of rapamycin complex 1
PI3K	Phosphatidyl inositol-3-kinase
PI3P	Phosphatidyl inositiol-3-phosphate
SNARE	Soluble N-ethylmaleimide-sensitive fusion attachment protein receptor
V-ATPase	Vacuolar adenosine triphosphatase
HSF	Heat shock factor
HSP	Heat shock protein
Snf1	Sucrose non-fermenting 1
AMPK	Adenosine monophosphate kinase
AMP	Adenosine monophosphate
ULK	Unc-51 like autophagy activating kinase
NAD	Nicotinamide adenosine dinucleotide
SIRT1	Sirtuin 1
TFEB	Transcription factor EB
LAMP1	Lysosome associated membrane protein 1
ZKSCAN3	Zinc finger with KRAB and SCAN domain 3
Ras	Rat Sarcoma
Akt	Protein kinase B
cAMP	Cyclic AMP
TSC	Tuberous sclerosis complex
CAMKKβ	Ca2+/calmodulin dependent protein kinase β
BECN1	Beclin 1
Fkh	Fork head
FOX	Fork head box
Hcm1	High-copy suppressor of calmodulin 1
Sesn3	Sestrin 3
MAP1LC3B	Microtubule associated protein 1 light chain 3 beta
GABARAPL1	γ-amino butyric acid type A receptor associated protein like 1
Rab7	Member RAS Oncogene family
ROS	Reactive oxygen species
RNS	Reactive nitrogen species
NRF2	Nuclear factor erythroid 2-related factor 2
Skn7	Supressor of Kre Null 7
ARE	Antioxidant response element
MAPK	Mitogen activated protein kinase
MAPKK	MAPK kinase
MAPKKK	MAPK kinase kinase
JNK	c-Jun N-terminal kinase
PKA	Protein kinase A
RAVE	Regulator of ATPase of vacuoles and endosomes
Rheb	Ras homolog enriched in brain
AXIN	Axis inhibitor
LKB1	Liver kinase B1
DUB	Deubiquitinating enzyme
GSK3β	Glycogen synthase kinase 3 beta
βCTF	Beta C-terminal fragment
TLN	Telencephalin
